# The (dis)engagement of mangrove forests and mangrove rice in academic and non-academic literature on Guinea-Bissau–a systematic review protocol

**DOI:** 10.1371/journal.pone.0284266

**Published:** 2023-04-13

**Authors:** Joana Sousa, Rita Campos, Orlando Mendes, Paula Duarte Lopes, Madalena Matias, Ana Paula Rosa, Raul Mendes Fernandes, Cristina Cruz, Bucar Indjai, Adilson Infande, Maira da Costa, Gonçalo Salvaterra, Juelson Lourenço, Dionísio Tavares, Djone Camala, Andrew Ainslie, Luís Catarino

**Affiliations:** 1 Centre for Social Studies, University of Coimbra (CES-UC), Coimbra, Portugal; 2 Instituto de Geografia e Ordenamento do Território, Centro de Estudos Geográficos, Universidade de Lisboa, Lisbon, Portugal; 3 Faculty of Economics, Centre for Social Studies, University of Coimbra, Coimbra, Portugal; 4 Faculdade de Ciências da Universidade de Lisboa, Centre for Ecology, Evolution and Environmental Changes, Lisbon, Portugal; 5 Universidade Amilcar Cabral, Bissau, Guinea-Bissau; 6 Instituto Nacional de Estudos e Pesquisa, Centro de Estudos Ambientais e Tecnologia Apropriada, Bissau (CEATA-INEP), Bissau, Guinea-Bissau; 7 Instituto Universitário de Lisboa (ISCTE-IUL), Lisbon, Portugal; 8 Centre for Research in Anthropology, ISCTE-IUL/ FCSH-UNL, Lisboa, Portugal; 9 Organização para a Defesa e Desenvolvimento das Zonas Húmidas (ODZH), Bissau, Guinea-Bissau; 10 University of Reading, Reading, United Kingdom; Xiamen University - Malaysia Campus: Xiamen University - Malaysia, MALAYSIA

## Abstract

**Background:**

Coastal areas in Guinea-Bissau and elsewhere in West Africa are bordered by mangrove forests. In several of these places, swaths of mangrove forest have been removed and the landscape has been technologically adapted for the production of mangrove rice–a regionally important staple. However, the effects of global warming, in particular sea-level rise, pose challenges to these socioecological environments. In this context, knowledge appears as an important resource and knowing what knowledge has been produced and which perspectives have guided that production may inform future responses to climate change. We have developed a systematic literature review protocol focusing on the main question: “How have mangrove forest and mangrove rice spaces been represented in the literature on Guinea-Bissau?” The main hypothesis is that although they occupy contiguous, interrelated and interactant spaces in coastal environments, mangrove forests and mangrove rice have been studied and analyzed independently in the literature.

**Methods:**

This is a protocol for conducting a systematic review that will include academic and non-academic literature in Portuguese, English and French. The academic literature will be retrieved from both Web of Science and Scopus using Boolean expressions. The non-academic literature will be accessed from relevant institutions, specialized libraries, and reference lists of previously selected items. Data extraction will follow a standard procedure based on an information sheet. Our analysis will be both qualitative (inductive and deductive coding, content analysis) and quantitative (word clouds, descriptive statistics and statistical testing).

**Discussion:**

This systematic review will provide information about the conceptual framework that has been produced through research, policymaking, and conservation and development programs in the management of coastal areas. This study will identify the limitations of previous approaches and contribute to both future research and strategies for planning adaptation to climate change. Finally, the outputs will add to broader debates about people-nature coexistence and climate change adaptation and mitigation.

## Introduction

The dichotomy of nature and society, well-founded in the epistemologies of the Global North, has been broadly described as narrowing the possibilities of knowing and engaging [1]. Critical approaches to nature and culture divide have been shaped by multi-species ethnographies [2], and notions like assemblage [3] or hybrid geographies [4], to only name a few, or the Amerindian perspectivism [5], which, like other worldviews from the Global South, have challenged the society-nature divide and brought possibilities to rethink coexistence. Worldviews from the margins can add valuable and historically overlooked perspectives to the plurality of analyses drawn about a world in need of urgent socioecological reformulation. In recent years, and still in development, the boundaries of nature are being questioned further in debates about the apocalypse, the “end of the world”, the “end of nature”, in the context of the current climatic crisis [6].

Climate change is affecting the planet’s different strata (atmosphere, biosphere, lithosphere, hydrosphere) and the way their interactions operate at the global scale [7]. There is broad consensus today that the increasing trends in the average global temperatures of the last century and the increase in the length of dry spells are affecting both ecosystems and ecosystem services, further accentuating the vulnerabilities of communities that depend heavily on these services [8]. It has been further recognized that the distribution of impacts is not equal or equitable around the world, as some people and other non-human beings have already been and will continue to be more severely affected than others. The asymmetries in disruption, harm, and death resulting from global warming arise from different biophysical conditions (e.g., low-lying areas, drylands, monsoon areas) as well as from historical and structural socioeconomic and political conditions that have created context-specific vulnerabilities which, in turn, limit capacity to respond to the effects of climatic changes. These heterogeneities have been developed considerably in the critique of the Anthropocene as a rigorous explanatory concept [6,9–12].

If it is true that these asymmetries have been consistently reported in the literature [13] and in international discourse [14,15], it is also true that insufficient and/or ineffective political effort has been exerted towards interrupting processes of vulnerabilization and eliminating the causes of climate change [16]. Therefore, it remains important to pay attention to places where the effects of global warming overlap with historical and political vulnerabilities and, from there, to examine baseline knowledge and global connections contributing to the responses to climate change. Recently, in the 27th United Nations climate change conference (COP27), the parties agreed on the need to “acknowledge the urgent and immediate need for new, additional, predictable and adequate financial resources to assist developing countries that are particularly vulnerable to the adverse effects of climate change in responding to economic and non-economic loss and damage associated with the adverse effects of climate change, including extreme weather events and slow onset events, especially in the context of ongoing and ex post (including rehabilitation, recovery and reconstruction) action” (p. 2) [15]. This is particularly important to countries like Guinea-Bissau and can potentially address problems specific to its coastal areas and to coastal societies.

Guinea-Bissau, located on the West African coast, is considered as one of the 38 Small Island Developing States (SIDS). Together with Comoros, Haiti, Kiribati, São Tomé and Príncipe, Solomon Islands, Timor-Leste and Tuvalu, Guinea-Bissau is both a SIDS and a Least Developed Countries (LDC) [17]. Each of the countries had a long-term history of colonization by imperial powers, with Guinea-Bissau, São Tomé e Príncipe and Timor-Leste having been submitted to Portuguese colonialism. All, with the notable exception of Haiti, only achieved internationally recognized independence in the mid to late 1970s.

Coastal Guinea-Bissau forms a unique standpoint vis-à-vis the place of nature and people in the context of climate change and might contribute to the pluralization of perspectives about nature and culture in a context of climate change.

### Context

The case of Guinea-Bissau provides a paradigmatic example of a country affected by overlapping layers of deep-rooted vulnerabilities. After a long period of commercial exploitation which depended on the slave trade (15-19^th^ centuries) [18], and another of colonial occupation characterized by coercion and ethnic division (early to mid 20th century) [19], Guinea-Bissau endured an eleven-year liberation struggle (1963–1974) [20]. Bissau-Guinean independence is celebrated on September 24, the day it was unilaterally declared in 1973 by the African Party for the Independence of Guinea and Cape Verde (PAIGC). In the less than 50 years since independence, Guinea-Bissau has experienced major political transitions, shifting from a single-party regime featuring a state-centered socialist model to a liberalized economic model with multi-party elections. This period was marked by a devastating civil war (1998–9) and an ongoing series of coups and coup attempts.

Today, the economy of Guinea-Bissau is largely based on agriculture, with the two most important crops being cashew nuts and rice. Cashew nut exports represent an important contribution to GDP (in 2019, agriculture, forestry and fishing represented 53.3% of the GDP (https://www.fao.org/faostat/en/#data/MK), and are primarily destined for India (in 2020 all 100,264 tons were shipped to India https://www.fao.org/faostat/en/#data/TM, https://www.fao.org/faostat/en/#data/TCL). Rice is the country-wide staple and the most culturally meaningful crop [21]. Beginning in the 1980s, the adoption of an exchange system between imported rice and cashew nuts incentivized the establishment of cashew orchards all over the country [22,23]. Importantly, though, local production of rice has been repeatedly reaffirmed as key to the country’s food security [24–26].

Rice is produced in upland rainfed areas (incorporating forest-fallow rotations), in freshwater swamps, and in coastal areas affected by tides. This third rice production practice typically occurs on land previously occupied by mangroves, leading to its designation as mangrove rice. Except maybe for the freshwater rice cultivation on private land concessions in the Geba River and in a few other places, the most productive rice system in Guinea-Bissau is the one installed in mangrove swamps [27]. Consequently, the possibility of the country achieving rice sovereignty largely depends on the production of rice in mangrove soil.

Mangrove rice producing systems rely on a technology-nature-ecology arrangement that creates an interface between mangrove forests and mangrove rice fields. Mangrove rice technologies include dikes that prevent saltwater from entering the paddies at high tide and that retain freshwater runoff. The maintenance of this interface depends on considerable labor, technology, and expert knowledge [28]. The existing infrastructure at this interface was severely affected by the colonial military attacks during the independence struggle [27]. The recuperation of damaged and/or abandoned mangrove rice technologies was then disincentivized by the structural readjustment of the economy beginning in 1985 [29]. This process has promoted cashew-rice exchange and further dependence on this cash crop. Challenges to the recovery of rice production have been further compounded by the rural exodus of youth to the cities [30]. Currently, mangrove rice farming is subject to major disruptive forces stemming from sea-level rise and other dire consequences of global warming. Thus, climate change, the long-term lingering effects of colonial exploitation and violence and short-sighted public policies adopted by Bissau-Guinean governments over the last few decades, have all contributed to important challenges to the reproduction of the modes of life and life systems of coastal farmers.

### Rationale

Several authors writing about the Anthropocene, especially when subjecting the concept to critical analysis, have provided convincing evidence that the effects of climate change must be understood through an approach that (i) reveals the unequal distribution of climate vulnerability and the historical, political and socioeconomic conditions that have created that vulnerability [6,9–12,31], and (ii) includes humans, non-human subjects and things as participants in world-making [32,33]. These two lenses have often been isolated from each other in the literature due to traditions of disciplinary siloing. This systematic review is based on the awareness raised by these recently developed perspectives, and applied to the study of a specific context. We will focus on a case, Guinea-Bissau, which as described above, has been heavily affected both by climate change and by structural political vulnerabilities. We will discuss the development of social and natural (dis)engagement and interdisciplinarity in academic and non-academic literature about the coastal environments in Guinea-Bissau.

Mangrove forests and mangrove rice fields in West Africa are part of an interface that spans from Gambia to Sierra Leone and that plays a crucial role in both food security and biodiversity conservation, two areas of concern that are typically studied in isolation. Mangrove forests have been variously described as crucial resources to coastal livelihoods [34,35], as nurseries to fish and crustacean species [36], as shields against climate change-related seawater flooding [37], and as carbon-rich ecosystems capable of generating carbon-revenues [38]. There are international initiatives for the protection of mangroves (UNESCO, Global Climate Fund, World Bank), and Guinea-Bissau has adopted national legislation and signed international agreements to that end (e.g., Ramsar convention). Mangrove rice farming, in its turn, has been studied in the context of the Atlantic history of tidal rice technology, which includes mangrove rice [39–41], and other studies have examined the social organization, agrobiodiversity and innovation of the so-called rice societies [28,42–48]. Thus, both mangrove forests and mangrove rice fields have received considerable attention from scholars and NGOs in Guinea-Bissau and internationally. However, previous studies have not discussed the relationship established between mangrove forests and mangrove rice in the literature in Guinea-Bissau or any other country, although both spaces are sometimes described in the same study. Examples of this are studies on the temporal analysis of land cover, ecosystem services or nature conservation, which take into consideration both mangrove rice and mangrove forests. This literature review will contribute to overcoming the literature gap that is given by a lack of studies about the ways in which these contiguous and interacting spaces have been conceived in the literature and, then, managed.

Our literature review will analyze the disciplinary lenses and voices in the study of mangrove forest-rice spaces and, from there, identify the conceptual and interdisciplinary perspectives that could be added to better understand these coastal landscapes. This contribution will add to the current framework of climate change literature to better understand the challenges imposed on coastal areas. The systematic review will also contribute to the development of better-suited strategies for planning climate change adaptation measures in the region and elsewhere.

### Research questions

To answer the overall research question: “How have mangrove forest and mangrove rice spaces been represented in the literature on Guinea-Bissau?”, the following sub-questions will be addressed by the proposed review:

**1. How have mangrove forests and mangrove rice been related to each other in academic and non-academic literature on Guinea-Bissau?**

Hypothesis 1a: Mangrove forests are not mentioned in studies focusing on mangrove rice;
Hypothesis 1b: Mangrove forests have been mainly studied by natural sciences (e.g., ecology, biology, agricultural sciences, conservation science);
Hypothesis 1c: Mangrove rice is not mentioned in studies focusing on mangrove forests;
Hypothesis 1d: Mangrove rice agriculture has been mainly studied by the social sciences (e.g., anthropology, sociology);
Hypothesis 1e: Studies that include both mangrove forests and mangrove rice present them as separate, discrete, and isolated [the opposite would be to represent them as connected and interacting].

**2. How has literature on mangrove forests and mangrove rice in Guinea-Bissau included the idea of climate change?**

Hypothesis 2a: Climate change is studied either in relation to rice production or in relation to mangrove forests, but not both;
Hypothesis 2b: Mangrove forests are portrayed as structures that protect against climate change;
Hypothesis 2c: Mangrove rice paddies are portrayed as places vulnerable to the effects of climate change.

## Methods

This review protocol follows the PRISMA-P guidelines, namely the preferred reporting items for systematic review and meta-analysis protocols (see S1 Checklist) [49]. We have also consulted the ROSES checklist for systematic review protocols [50].

Our review will include academic and non-academic literature in three languages: Portuguese, English and French. Portuguese is the official language in Guinea-Bissau and is frequently used in reports and non-indexed journals published in the country. Several anglophone and francophone scholars have published work on Guinea-Bissau and several Bissau-Guinean and international scholars have also published in English and French, therefore we found it relevant to also include these languages. French, in particular, is the dominant language in West Africa and especially in the countries bordering Guinea-Bissau. The literature in French allows us to follow knowledge traditions potentially relevant to Guinea-Bissau.

**Fig 1:**
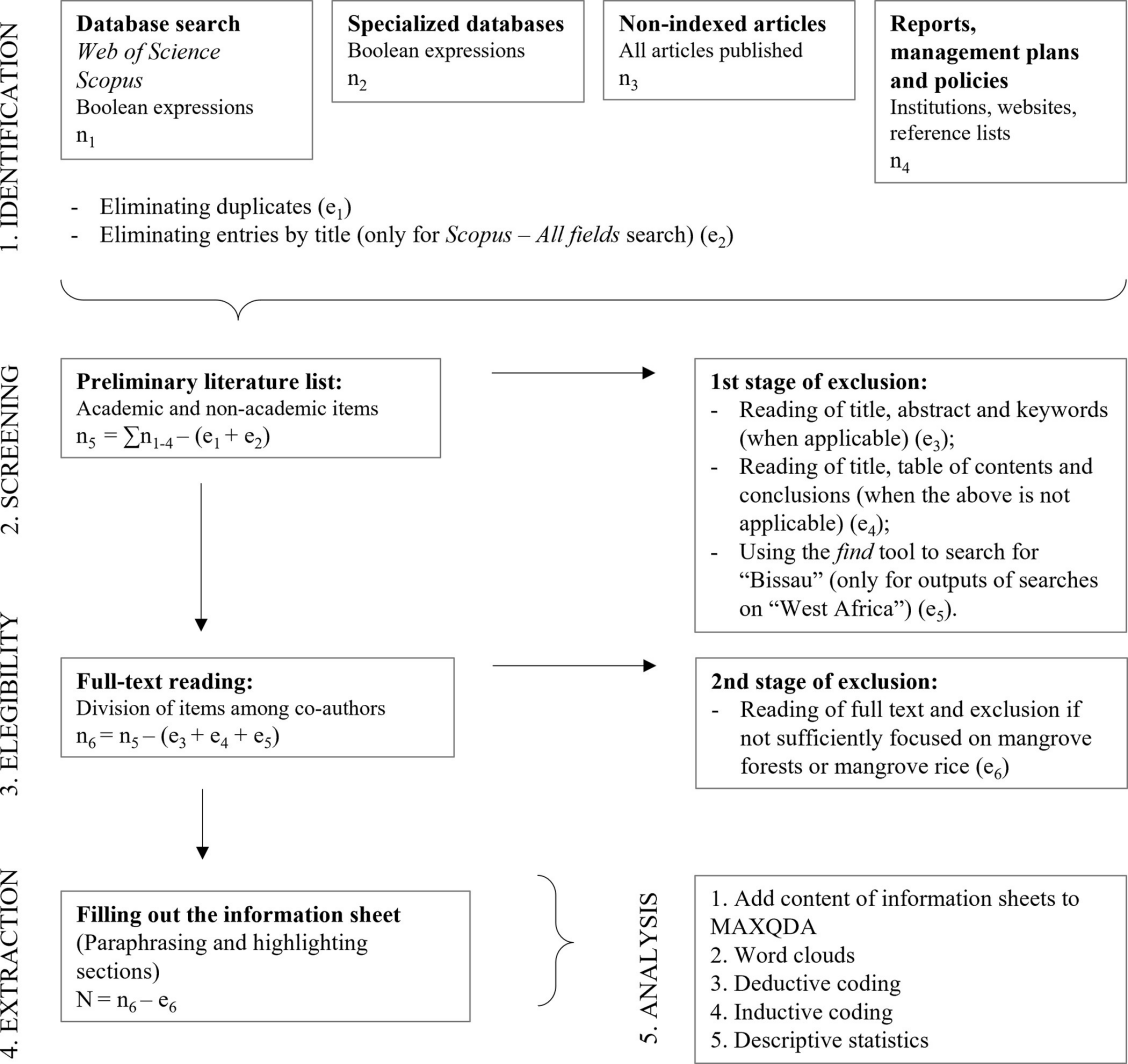
Phases of the review process from identification to analysis.

The review will comply with a research process detailed in five phases (see Fig 1).

### Search strategy

For the academic and peer-review literature (articles, books, book chapters) we will use Boolean expressions (Table 1) and conduct searches on both *Web of Science* and *Scopus*.

**Table 1:** Keywords and respective functions.

	Function
mangrov* AND/OR (rice* OR paddy) (mangal AND/OR arroz) (mangrove AND/OR riz)	Defines the objects of study. AND/OR defines the nature of their relationship (if any).
Bissau OR Bissao	Defines the geography of the review. “Bissau” is the part of the name of the country in Portuguese, English and French. “Bissao” is sometimes used in French.
“West Africa” “África Ocidental” “Afrique de l’Ouest” OR “Ouest Africaine”	
“climat*” OR “warm*” “clima*” OR “aquecimento” “climatique” OR “réchauff*”	Defines the climate change focus of the analyses under review.

In *Web of Science*, we will use the search option for *All fields*. This option includes titles, abstracts and keywords (which is equivalent to a TAK or a topic-based search) plus the index terms automatically generated from the titles of cited articles (keywords plus) as material to be surveyed. In Scopus we will focus on the search by topic (Title, Abstract, Keywords). We will also use the option *All fields*, which in this case considers the whole content of the literature list. As explained below, this will be used to a lesser extent.

For answering Research Question 1, we will start with a search for (1) mangrov* AND (rice* OR paddy) AND Bissau to retrieve articles that potentially focus on both mangroves and rice fields in the context of Guinea-Bissau. We will then proceed with second and third searches to retrieve articles focusing either on mangroves or on mangrove rice in Guinea-Bissau, respectively (2) mangrov* AND Bissau and (3) (rice* OR paddy) AND Bissau.

Potentially, Guinea-Bissau has been analyzed in studies with a regional focus in which the term “Bissau” may not have been included in the elements surveyed by *Web of Science* or *Scopus*. Therefore, we will make fourth, fifth and sixth searches with the same Boolean expression named above but replacing “Bissau” with “West Africa”: (4) mangrov* AND (rice* OR paddy) AND “West Africa”, (5) mangrov* AND “West Africa” and (6) (rice* OR paddy) AND “West Africa”.

In order to address Research Question 2, we will add “AND (climat* OR warm*)” to the expressions described above and repeat the six searches, which will provide the outputs for the 7–12 searches having a regional focus.

We will consider literature published since the period of independence (1973–1974) to 2023. Including the colonial period would direct our search to the archives, which would be time consuming and requires a different methodology.

For the qualitative search of non-academic and non-indexed academic literature we will also include documents in the three languages. The reading items will be gathered by means of three approaches.

We have compiled a comprehensive list of institutions, programs, libraries, and specialized websites that will be useful for carrying out the search (Table 2). First, we will visit the websites of international organizations working in Guinea-Bissau (Table 2) and retrieve any accessible documents (reports, action plans, consultancies) on mangrove and/or mangrove rice in Guinea-Bissau or West Africa. Second, we will contact institutions and people who have carried out studies and consultancies in Guinea-Bissau and ask them to share with us their published or released work. Third, we will access papers published in non-indexed journals in Guinea-Bissau (*Soronda*, *Boletim de Informação Socioeconómica*, *Boletim de Informação Científica e Técnica*, *Matu malgos*, *Palmeirinha*, *Katchu martel*, *Sintidus)* either by retrieving them online or by directly contacting authors or visiting libraries (e.g, INEP library, CIDAC). Journals published in Portugal and Brazil include, for example, *Etnográfica*, *Cadernos de Estudos Africanos*, *Revista Crítica de Ciências Sociais*, *Revista Internacional em Língua Portuguesa*, *Africa*: *Revista do Centro de Estudos Africanos da Universidade de São Paulo*. Other publications can be retrieved from the Arquivo Científico Tropical Digital (ACTD) organized by Instituto de Investigação Científica Tropical (Portugal) and from the Cahiers Agricultures (https://www.cahiersagricultures.fr/https://www.cahiersagricultures.fr/) and CIRAD online publications (https://revues.cirad.fr). We will also check publications by CODESRIA in Senegal. Dissertations will be accessed from online databases in Portugal (https://www.rcaap.pt/), Brazil (Biblioteca Digital Brasileira de Teses e Dissertações, BDTD: https://bdtd.ibict.br/vufind/) the United Kingdom (British library: https://ethos.bl.uk/), USA (EBSCO: https://www.ebsco.com/products/research-databases/ebsco-open-dissertations), and worldwide (Open Access—Theses and Dissertation: https://oatd.org/).

**Table 2:** List of institutions and specialized websites.

Multilateral institutions	International NGOs	Specialized libraries and/or databases
Food and Agriculture Organization (FAO) United Nations Development Programme (UNDP) World Food Programme (WFP) European Union International Union for Conservation of Nature (IUCN) African Development Bank World Bank IMF CGIAR	Instituto Camões SwissAid USAID Association VIDA Swedish Cooperation (ASDI) Instituto Marquês Valle Flôr Universel SNV Wetlands International ADPP Lvia Bosque y Comunidad FIDA Caritas FEC GDZ	Memória de Africa Africa Catalogue Fundação Mário Soares CIDAC

Finally, we will use the literature lists of the relevant identified articles in order to identify other relevant literature. Titles that seem relevant and can be accessed will be added to the reading list. The inclusion of gray literature will be finalized when a saturation of the items that appear in the literature lists in the peer-reviewed articles consulted is achieved.

All identified literature will be imported to Zotero 6.0.10 for management and exclusion of duplicates.

### Selection criteria

First, the outcomes of all searches will be cross-checked to eliminate duplicates. Then, the title, abstract and keywords of the outcomes from Web of Science and Scopus focused on Guinea-Bissau (1–3 and 7–9) will be read and items that do not engage with mangroves or mangrove rice in Guinea-Bissau will be discarded. We will perform a close reading of selected papers and if the argument does not engage with either mangroves or mangrove rice-related themes, papers will be discarded. For the outcomes of items that resulted from searches on “West Africa” we will add a preliminary step. First, we will check if the term “Bissau” is mentioned in the text. If not, the document is discarded. Then, we will adopt the same procedure used for the search with a focus on “Bissau.” This will also be the procedure followed for all non-academic and non-indexed literature.

The Scopus search based on *All fields*, which includes the whole literature list, will not be considered for the searches (5) Mangrov* AND “West Africa” nor for (6) (Rice* OR paddy) AND “West Africa”. These searches would unnecessarily broaden the field to include thousands of outcomes that (i) are not connected to the realities of Guinea-Bissau (the term “Bissau” is not included in the search) and (ii) potentially are not even connected to mangrove rice or mangroves (since these terms may appear in literature lists without having been mentioned in the main text). We will maintain a record of the reasons for excluding literature items at all stages of the review.

### Data extraction

Selected literature will be divided among the authors for close reading. As individual texts are read, the review team will highlight important sections related to the two research questions. Then, the relevant information will be extracted from each text and included in a document following a completed information summary sheet addressing: (1) the journal (in case of articles) or publication, (2) the home institution of the first author, (3) the countries where authors are based, (4) the language the text was composed in, (5) the focus on mangroves/rice/both, (6) aspects of mangroves/rice/both that are under study, (7) the relationships established between mangrove forests and rice fields (if any), (8) references to climate change in relation to rice and/or mangroves, (9) the methodological approach, (10) the main argument and conclusions, (11) baseline concepts used, (12) policymaking and/or future research recommendations (if any), (13) critical analysis of the paper, and (14) references to specific institutions, projects, cases or other readings.

Each author will be responsible for a set of selected texts and will share completed information summary sheets with the rest of the team. These information sheets are expected to include direct citations of important paragraphs and paraphrasing explaining the major ideas and contributions of the paper (specially for points 5–13 mentioned above). Each text and information sheet will be validated by another member of the review team. Should the outcomes differ substantially, the process for data extraction will be further clarified and discussed in monthly meetings with the review team, and the procedure reformulated accordingly.

### Risk of bias

Two types of risk of bias are important to consider in this protocol, one refers to the individual studies included in the systematic review, and the other to the procedures defined for the review, which can potentially lead to a risk of bias due to missing results [51].

Studies to be included in the review encompass articles published in peer-review and indexed journals, which theoretically had a team of editors and reviewers prompting authors to report their methods transparently [51]. The review will also include articles published in non-indexed journals and reports. Reports to be selected are limited to texts released by international organizations and/or referenced in papers published in indexed journals. Despite their structural differences, all articles and reports will be subjected to individual assessment for risk of bias.

The risk of bias assessment will be carried out independently by two authors. To guarantee that the criteria for assessing bias will be applied consistently, both authors will conduct a previous evaluation of a sample consisting of three articles and three reports. Any disagreement or discrepancies in the individual assessment for risk bias will be discussed and resolved. Articles and reports will be rated for low risk of bias and high risk of bias according to the following criteria: (i) bias due to detail about study design and methods, (ii) bias due to missing data and/or inconsistency of results (iii) bias due to unexplained heterogeneity and/or overgeneralization. A text that meets two of the criteria above will be classified as high risk of bias and a text that meets one criterion or less will be classified as low risk of bias.

Risk of bias assessments will be included in the review by following a stratified analysis according to the two categories of risk of bias considered. The review will mainly focus on studies with a low risk of bias and information extracted from studies with a high risk of bias will be included in the results as additional information and will be adequately signposted.

A measure of saturation of the non-indexed papers and reports will be given by the analysis of the literature lists of articles previously read and will be used to assess the risk of bias due to missing results.

The authors have no conflict of interest. An account of the limitations of the study will consider the sources of bias identified above and others that might be found during the review process [52].

### Protocol registration

The review protocol is registered with The Open Science Framework (OSF; registration DOI: 10.17605/OSF.IO/P8TBQ). The authors will document any amendments to the protocol in the OSF record.

### Data analysis

Information extracted from the review will mainly be analyzed qualitatively although we will also perform some quantitative analysis. We will use content analysis for the qualitative analysis. The information sheets produced during data extraction, as well as the highlighted sections of the texts, will be coded in MAXQDA 2020.

For the coding process, we will use both inductive and deductive coding. The former will capture the content developed by the different readers of the review team and expressed in the information sheets, which will be based on the attribution of codes according to each of the 13 points that guided data extraction. Deductive coding will follow a grounded theory approach, which will be conducted on the highlighted sections of the texts included in the final reading list and will be based on open coding, axial coding, and selective coding [53]. Open coding constitutes the first abstraction step in which the first set of concepts is identified and (re-)labeled from the highlighted sections and the information sheets. Then, axial coding aims at identifying the relationships between concepts previously identified, which are grouped into categories. Selective coding will then be used to integrate and refine the categories that were identified and develop relationships between them. This is the phase most prone to the elaboration of theoretical insights that potentially can explain the conceptual structures that have guided the production of knowledge in the literature.

The presentation of the content analysis will be organized in dedicated sections in the report manuscript, arranged according to the main themes that arise from the procedure explained above. An initial step in the quantitative analysis is to produce a database of different variables classifying each text. For example, the responses to points 1–5 on the information sheets (journal, institution of the first author, countries where authors are based, language, focus on mangrove/rice/both) would be entered for each text. Additionally, post-categorization of information will be carried out, including, for example, the disciplinary frame, policy-making recommendations, research recommendations, suggested strategies for climate change adaptation and/or mitigation.

MAXQDA 2022 will be used to provide word clouds (a quantitative visualization tool). Simple statistical descriptors (means and proportions, time distribution of publications) will be used to illustrate data distribution. Then, chi-square tests will be performed to assess the relationship between the categorical variables named above. All hypotheses, 1a-e and 2a-e (see Research questions section above), will be tested by 2x2 chi-square tests, in which input data is frequency values extracted from a presence-absence table. Hypotheses 1e, 2b and 2c will also be analysed by means of qualitative coding in MAXQDA, as described above. Tests will be performed in SPSS v.20 and we will assume a threshold of statistical significance of p<0,01.

## Discussion

This study analyses the conceptual frames and disciplinary and interdisciplinary lenses used to understand coastal areas in Guinea-Bissau, particularly the interface of mangrove forests and rice fields. A comparison between studies focusing on rice, studies focusing on mangroves and studies considering both, will guide the discussion. We will characterise the relationship established between the mangrove and rice realms historically and identify what were the triggers and/or lenses that allowed for them to be studied as related units, if any. The dichotomy of nature and society has been described as limited to understand socioecological complexities [1]. Critical approaches to nature have led to theoretical developments based on a socially constructed nature. Lately, the boundaries of nature are being questioned further in the context of the current climatic crisis [54]. To engage with these recent debates, we will give special attention to the ways in which mangrove forests and mangrove rice figure in the narratives and knowledge produced about climate change in coastal Guinea-Bissau. Finally, the review will promote the dialogue of political, social and ecological perspectives on current controversies about climate change, conservation, development and food security.

## Supporting information

S1 Checklist Preferred reporting items for systematic review and meta-analysis protocols. (DOC)
